# Supplementary material for: A Likelihood Approach for Real-Time Calibration of Stochastic Compartmental Epidemic Models
Source: PLoS Comput Biol. 2017 Jan 17;13(1):e1005257. doi: 10.1371/journal.pcbi.1005257 (PMC5240920; doi:10.1371/journal.pcbi.1005257)
Supplement: S1 File — (TAR.GZ) [file pcbi.1005257.s014.tar.gz › HSPH_Online-SI-Revision/MSS10/n10-severe/n10-severe_NEW2_table0.pdf]

1	newly 3 weeks spec	422.	498.15	{465., 528.}	18.045	0.0713805	newly 3 weeks cumul	1572.	1737.7	{1678., 1802.}	10.5407	0.0433454	newly 5 weeks spec	319.	324.46	{295., 349.}	5.46708	0.023371	newly 5 weeks cumul	2230.	2468.75	{2375., 2565.}	10.7063	0.0440059	newly 51 weeks cumul	3236.	3505.55	{3381., 3634.}	8.32973	0.0345825
2	newly 3 weeks spec	559.	312.61	{249., 369.}	44.0769	0.257042	newly 3 weeks cumul	1850.	1397.79	{1200., 1574.}	24.4968	0.124592	newly 5 weeks spec	307.	123.31	{82., 165.}	59.8339	0.409746	newly 5 weeks cumul	2596.	1721.42	{1491., 1945.}	33.6895	0.180675	newly 53 weeks cumul	3551.	1905.07	{1655., 2155.}	46.3512	0.272432
3	newly 3 weeks spec	372.	526.55	{339., 601.}	45.5403	0.161918	newly 3 weeks cumul	1462.	1776.63	{1376., 1913.}	23.9432	0.0931158	newly 5 weeks spec	176.	372.33	{159., 443.}	117.528	0.329817	newly 5 weeks cumul	1920.	2600.08	{1722., 2839.}	38.6115	0.140857	newly 55 weeks cumul	2258.	3943.15	{2079., 4422.}	78.2139	0.244655
4	newly 3 weeks spec	431.	355.95	{266., 464.}	20.3828	0.103098	newly 3 weeks cumul	1668.	1483.29	{1251., 1727.}	13.0546	0.0627185	newly 5 weeks spec	272.	164.14	{105., 234.}	39.9191	0.238646	newly 5 weeks cumul	2316.	1895.25	{1564., 2272.}	19.1006	0.0955645	newly 50 weeks cumul	2990.	2195.83	{1700., 2830.}	26.6525	0.14077
5	newly 3 weeks spec	395.	336.22	{297., 381.}	15.1291	0.0733727	newly 3 weeks cumul	1512.	1292.	{1172., 1397.}	14.5503	0.0692282	newly 5 weeks spec	274.	177.5	{141., 216.}	35.219	0.19422	newly 5 weeks cumul	2125.	1716.51	{1558., 1856.}	19.2231	0.0937893	newly 50 weeks cumul	3018.	2124.77	{1941., 2317.}	29.5968	0.15361
6	newly 3 weeks spec	500.	228.4	{88., 588.}	60.944	0.465232	newly 3 weeks cumul	1731.	1091.43	{665., 1925.}	41.1525	0.251194	newly 5 weeks spec	216.	101.45	{11., 409.}	84.5046	0.818025	newly 5 weeks cumul	2283.	1342.16	{714., 2849.}	49.9921	0.317752	newly 51 weeks cumul	2710.	1617.13	{719., 4233.}	60.421	0.384421
7	newly 3 weeks spec	266.	232.68	{196., 274.}	14.1579	0.072974	newly 3 weeks cumul	943.	869.49	{759., 983.}	10.0286	0.0480536	newly 5 weeks spec	142.	137.78	{104., 167.}	14.5634	0.0724274	newly 5 weeks cumul	1279.	1187.44	{1038., 1345.}	9.55278	0.046486	newly 50 weeks cumul	1805.	1577.81	{1409., 1763.}	12.8892	0.0648062
8	newly 3 weeks spec	348.	598.49	{464., 669.}	72.2615	0.231463	newly 3 weeks cumul	1582.	2040.95	{1750., 2193.}	29.8767	0.112281	newly 5 weeks spec	203.	400.04	{268., 465.}	99.3498	0.293084	newly 5 weeks cumul	2074.	2940.56	{2370., 3183.}	42.9807	0.153244	newly 54 weeks cumul	2481.	4179.28	{2988., 4598.}	69.8896	0.225467
9	newly 3 weeks spec	571.	234.89	{139., 319.}	58.9229	0.413904	newly 3 weeks cumul	2047.	1270.42	{884., 1580.}	38.2013	0.221333	newly 5 weeks spec	361.	67.32	{28., 108.}	81.3518	0.805451	newly 5 weeks cumul	2908.	1469.37	{1061., 1758.}	49.4715	0.310451	newly 58 weeks cumul	3573.	1542.08	{1087., 1846.}	56.8408	0.380367
10	newly 3 weeks spec	479.	277.16	{221., 325.}	42.1378	0.242628	newly 3 weeks cumul	1759.	1361.15	{1123., 1579.}	22.6941	0.115441	newly 5 weeks spec	209.	93.95	{65., 119.}	55.0478	0.36004	newly 5 weeks cumul	2339.	1617.63	{1292., 1855.}	30.841	0.163769	newly 58 weeks cumul	2666.	1736.19	{1414., 1980.}	34.8766	0.189498
11	newly 3 weeks spec	358.	209.4	{103., 298.}	41.8547	0.263458	newly 3 weeks cumul	1919.	1324.9	{674., 1834.}	31.2715	0.191736	newly 5 weeks spec	90.	46.79	{13., 94.}	52.3889	0.425616	newly 5 weeks cumul	2152.	1471.76	{774., 1977.}	31.8457	0.191046	newly 57 weeks cumul	2254.	1507.88	{813., 1998.}	33.2023	0.19766
12	newly 3 weeks spec	587.	486.07	{381., 562.}	21.8893	0.112307	newly 3 weeks cumul	1997.	1898.55	{1534., 2186.}	10.8277	0.0514695	newly 5 weeks spec	284.	217.54	{150., 254.}	36.1479	0.191495	newly 5 weeks cumul	2683.	2452.36	{2045., 2740.}	14.4204	0.068817	newly 57 weeks cumul	3203.	2834.57	{2336., 3075.}	20.9145	0.0980715
13	newly 3 weeks spec	574.	467.46	{285., 666.}	29.2613	0.155163	newly 3 weeks cumul	2148.	1931.67	{1416., 2385.}	18.601	0.0895967	newly 5 weeks spec	276.	219.9	{98., 340.}	43.2536	0.244784	newly 5 weeks cumul	2826.	2477.68	{1747., 3148.}	23.3015	0.114424	newly 54 weeks cumul	3484.	2916.34	{1892., 3723.}	29.7968	0.151135
14	newly 3 weeks spec	353.	287.9	{241., 335.}	19.1785	0.0954208	newly 3 weeks cumul	1439.	1448.27	{1240., 1659.}	8.30368	0.0361021	newly 5 weeks spec	163.	101.26	{71., 135.}	37.8773	0.219182	newly 5 weeks cumul	1856.	1721.95	{1490., 1960.}	9.85075	0.0456199	newly 56 weeks cumul	2090.	1856.08	{1645., 2103.}	12.0048	0.0566918
15	newly 3 weeks spec	511.	331.	{269., 392.}	35.225	0.192709	newly 3 weeks cumul	2278.	1733.92	{1507., 1918.}	23.8841	0.120725	newly 5 weeks spec	197.	100.36	{69., 123.}	49.5228	0.30597	newly 5 weeks cumul	2829.	2018.97	{1756., 2235.}	28.6331	0.148415	newly 60 weeks cumul	3055.	2130.46	{1888., 2366.}	30.2632	0.158367
16	newly 3 weeks spec	366.	628.26	{599., 667.}	72.0109	0.233236	newly 3 weeks cumul	1694.	2278.01	{2265., 2364.}	35.3571	0.131092	newly 5 weeks spec	184.	403.52	{381., 447.}	119.87	0.337318	newly 5 weeks cumul	2137.	3194.9	{3212., 3309.}	50.2368	0.175721	newly 56 weeks cumul	2454.	4545.1	{4608., 4730.}	85.8346	0.266283
17	newly 3 weeks spec	538.	353.93	{138., 468.}	34.2138	0.22928	newly 3 weeks cumul	1689.	1348.34	{762., 1653.}	20.3505	0.119739	newly 5 weeks spec	353.	193.96	{50., 283.}	45.0538	0.341335	newly 5 weeks cumul	2470.	1810.5	{916., 2276.}	26.7004	0.162725	newly 56 weeks cumul	3959.	2246.2	{977., 2912.}	43.2635	0.283712
18	newly 3 weeks spec	567.	367.51	{323., 409.}	35.1834	0.190076	newly 3 weeks cumul	1753.	1383.64	{1272., 1491.}	21.0702	0.103647	newly 5 weeks spec	394.	198.73	{167., 233.}	49.5609	0.301214	newly 5 weeks cumul	2605.	1855.95	{1697., 2012.}	28.7543	0.148062	newly 54 weeks cumul	3791.	2286.05	{2124., 2470.}	39.698	0.220511
19	newly 3 weeks spec	442.	225.03	{181., 268.}	49.0882	0.298714	newly 3 weeks cumul	1574.	1136.7	{972., 1289.}	27.7827	0.143843	newly 5 weeks spec	313.	79.39	{48., 111.}	74.6358	0.615401	newly 5 weeks cumul	2213.	1348.65	{1170., 1518.}	39.0578	0.217478	newly 50 weeks cumul	2944.	1445.33	{1230., 1637.}	50.9059	0.311554
20	newly 3 weeks spec	441.	271.37	{171., 376.}	38.4649	0.233491	newly 3 weeks cumul	1300.	1037.59	{766., 1281.}	21.1162	0.113135	newly 5 weeks spec	373.	148.57	{61., 247.}	60.1689	0.484355	newly 5 weeks cumul	2074.	1390.21	{952., 1831.}	32.9696	0.189093	newly 50 weeks cumul	3655.	1785.58	{1045., 2546.}	51.1469	0.337629
21	newly 3 weeks spec	460.	313.23	{264., 356.}	31.9065	0.170093	newly 3 weeks cumul	1920.	1512.08	{1340., 1681.}	21.2458	0.105721	newly 5 weeks spec	202.	106.66	{73., 142.}	47.198	0.290551	newly 5 weeks cumul	2437.	1804.62	{1599., 1989.}	25.9491	0.132201	newly 58 weeks cumul	2952.	1937.87	{1717., 2132.}	34.354	0.1844
22	newly 3 weeks spec	509.	528.51	{215., 645.}	28.0138	0.146682	newly 3 weeks cumul	1513.	1674.84	{969., 1918.}	24.1362	0.107051	newly 5 weeks spec	221.	419.4	{71., 530.}	115.557	0.385325	newly 5 weeks cumul	2121.	2578.68	{1271., 3018.}	38.7449	0.162725	newly 56 weeks cumul	2643.	4399.23	{1394., 5307.}	86.3553	0.29484
23	newly 3 weeks spec	421.	286.51	{226., 336.}	31.9454	0.172537	newly 3 weeks cumul	1389.	1129.68	{965., 1297.}	18.6695	0.0925589	newly 5 weeks spec	276.	145.7	{104., 191.}	47.2101	0.288269	newly 5 weeks cumul	2005.	1483.49	{1224., 1696.}	26.0105	0.133816	newly 55 weeks cumul	2915.	1805.5	{1549., 2090.}	38.0617	0.21091
24	newly 3 weeks spec	587.	418.81	{346., 496.}	28.9659	0.152204	newly 3 weeks cumul	2481.	2070.56	{1832., 2307.}	16.9109	0.0821632	newly 5 weeks spec	276.	131.62	{94., 170.}	52.3116	0.333687	newly 5 weeks cumul	3169.	2445.01	{2178., 2733.}	22.9962	0.115199	newly 56 weeks cumul	3630.	2585.12	{2279., 2877.}	28.7846	0.149298
25	newly 3 weeks spec	500.	301.39	{176., 407.}	40.078	0.24423	newly 3 weeks cumul	2503.	2021.88	{1547., 2442.}	20.1726	0.108542	newly 5 weeks spec	93.	56.98	{9., 112.}	49.6559	0.403005	newly 5 weeks cumul	2800.	2212.19	{1678., 2660.}	21.9604	0.118357	newly 59 weeks cumul	2905.	2254.03	{1758., 2698.}	23.4255	0.126753
26	newly 3 weeks spec	372.	180.45	{99., 255.}	51.4919	0.334276	newly 3 weeks cumul	1508.	974.92	{688., 1230.}	35.3501	0.200375	newly 5 weeks spec	264.	54.69	{13., 105.}	79.2841	0.793033	newly 5 weeks cumul	2071.	1130.96	{858., 1397.}	45.3906	0.273506	newly 58 weeks cumul	2508.	1195.67	{866., 1536.}	52.3258	0.333378
27	newly 3 weeks spec	638.	297.97	{155., 824.}	62.3871	0.435977	newly 3 weeks cumul	2129.	1474.17	{965., 2801.}	38.4796	0.212211	newly 5 weeks spec	370.	116.82	{30., 550.}	81.8378	0.784096	newly 5 weeks cumul	2994.	1773.26	{1136., 4092.}	49.7896	0.29802	newly 59 weeks cumul	3855.	2001.34	{1199., 5645.}	59.4241	0.383835
28	newly 3 weeks spec	519.	950.74	{914., 987.}	83.1869	0.262701	newly 3 weeks cumul	1948.	3086.99	{3034., 3140.}	58.4697	0.199906	newly 5 weeks spec	219.	644.87	{613., 676.}	194.461	0.468695	newly 5 weeks cumul	2468.	4533.49	{4465., 4594.}	83.6908	0.264062	newly 58 weeks cumul	2735.	6645.83	{6598., 6690.}	142.992	0.385586
29	newly 3 weeks spec	430.	409.39	{342., 483.}	9.8814	0.046109	newly 3 weeks cumul	2046.	2108.53	{1860., 2302.}	7.54008	0.0322141	newly 5 weeks spec	157.	121.19	{92., 151.}	24.7834	0.129964	newly 5 weeks cumul	2479.	2457.53	{2180., 2675.}	6.27955	0.0278693	newly 60 weeks cumul	2601.	2587.26	{2340., 2784.}	5.87236	0.0259321
30	newly 3 weeks spec	581.	675.95	{535., 745.}	20.1704	0.0809926	newly 3 weeks cumul	2678.	2949.99	{2567., 3146.}	12.2013	0.0502328	newly 5 weeks spec	176.	233.13	{172., 284.}	38.1989	0.142234	newly 5 weeks cumul	3250.	3591.49	{3003., 3819.}	13.2957	0.0549091	newly 60 weeks cumul	3417.	3879.95	{3143., 4130.}	16.3614	0.066499
31	newly 3 weeks spec	369.	132.47	{84., 183.}	64.1003	0.47253	newly 3 weeks cumul	1285.	822.64	{582., 1063.}	36.1463	0.20837	newly 5 weeks spec	236.	33.65	{8., 58.}	85.7415	0.958647	newly 5 weeks cumul	1779.	924.46	{651., 1188.}	48.0349	0.29755	newly 57 weeks cumul	2329.	956.63	{699., 1244.}	58.9253	0.399589
32	newly 3 weeks spec	297.	206.89	{152., 262.}	30.3603	0.166774	newly 3 weeks cumul	969.	818.08	{697., 958.}	16.0784	0.0796521	newly 5 weeks spec	208.	109.84	{63., 157.}	47.1923	0.307905	newly 5 weeks cumul	1426.	1081.52	{903., 1300.}	24.1571	0.125686	newly 50 weeks cumul	2189.	1370.7	{1051., 1705.}	37.3824	0.212278
33	newly 3 weeks spec	452.	308.76	{236., 402.}	31.9513	0.176125	newly 3 weeks cumul	1482.	1154.67	{974., 1376.}	22.2018	0.112895	newly 5 weeks spec	292.	178.89	{112., 268.}	39.2774	0.235404	newly 5 weeks cumul	2155.	1568.44	{1289., 1971.}	27.2817	0.143781	newly 50 weeks cumul	3095.	2038.82	{1578., 2840.}	34.2359	0.191687
34	newly 3 weeks spec	392.	328.02	{292., 368.}	16.3214	0.0789885	newly 3 weeks cumul	1312.	1202.01	{1101., 1299.}	8.51753	0.0393129	newly 5 weeks spec	229.	192.9	{164., 220.}	16.1397	0.0788377	newly 5 weeks cumul	1839.	1647.13	{1514., 1788.}	10.4606	0.0487116	newly 50 weeks cumul	2740.	2204.6	{2053., 2371.}	19.5401	0.0950546
35	newly 3 weeks spec	441.	825.48	{792., 866.}	87.1837	0.271995	newly 3 weeks cumul	1805.	2638.83	{2586., 2694.}	46.1956	0.164877	newly 5 weeks spec	239.	584.38	{550., 621.}	144.51	0.387781	newly 5 weeks cumul	2353.	3930.32	{3867., 3995.}	67.0344	0.222765	newly 57 weeks cumul	2899.	5975.6	{5905., 6038.}	106.126	0.314118
36	newly 3 weeks spec	507.	419.56	{299., 514.}	18.4418	0.0969509	newly 3 weeks cumul	1570.	1463.99	{1179., 1687.}	11.035	0.052746	newly 5 weeks spec	332.	262.69	{147., 354.}	24.2982	0.143067	newly 5 weeks cumul	2346.	2060.93	{1577., 2441.}	14.2877	0.0725796	newly 53 weeks cumul	3516.	2813.28	{1907., 3482.}	20.1035	0.110523
37	newly 3 weeks spec	390.	162.73	{91., 246.}	58.2744	0.416491	newly 3 weeks cumul	1418.	875.44	{488., 1148.}	38.2623	0.232101	newly 5 weeks spec	278.	51.55	{18., 92.}	81.4568	0.849175	newly 5 weeks cumul	2082.	1017.96	{602., 1352.}	51.1066	0.332071	newly 56 weeks cumul	2812.	1073.78	{618., 1412.}	61.8144	0.438676
38	newly 3 weeks spec	545.	653.21	{601., 706.}	19.855	0.0778448	newly 3 weeks cumul	1921.	2164.7	{2069., 2246.}	12.6861	0.0515945	newly 5 weeks spec	367.	442.62	{408., 475.}	20.6049	0.0804144	newly 5 weeks cumul	2763.	3156.27	{3022., 3259.}	14.2334	0.0575045	newly 56 weeks cumul	3774.	4514.84	{4366., 4666.}	19.6301	0.0775741
39	newly 3 weeks spec	417.	347.14	{252., 430.}	18.705	0.0963623	newly 3 weeks cumul	1435.	1269.19	{1037., 1481.}	12.9066	0.0631882	newly 5 weeks spec	292.	205.17	{127., 283.}	30.0377	0.172596	newly 5 weeks cumul	2099.	1746.67	{1377., 2095.}	17.1591	0.0872731	newly 52 weeks cumul	3353.	2312.74	{1749., 2904.}	31.0248	0.171231
40	newly 3 weeks spec	501.	379.99	{291., 565.}	28.6567	0.157742	newly 3 weeks cumul	1605.	1399.4	{1188., 1816.}	17.9327	0.0865828	newly 5 weeks spec	314.	223.13	{150., 386.}	37.4045	0.227311	newly 5 weeks cumul	2336.	1910.13	{1616., 2704.}	23.7924	0.12036	newly 52 weeks cumul	3452.	2470.95	{1918., 4060.}	34.3253	0.188945
41	newly 3 weeks spec	586.	494.79	{430., 558.}	15.6263	0.0757978	newly 3 weeks cumul	2370.	2220.37	{1939., 2486.}	8.55485	0.0395146	newly 5 weeks spec	259.	168.11	{130., 212.}	35.0927	0.195459	newly 5 weeks cumul	2995.	2687.89	{2441., 2969.}	10.5165	0.0493409	newly 60 weeks cumul	3338.	2882.4	{2647., 3163.}	13.6489	0.0646762
42	newly 3 weeks spec	574.	335.33	{271., 390.}	41.5801	0.237769	newly 3 weeks cumul	2136.	1574.57	{1353., 1753.}	26.2842	0.134635	newly 5 weeks spec	240.	118.26	{90., 154.}	50.725	0.317794	newly 5 weeks cumul	2747.	1893.49	{1654., 2111.}	31.0706	0.163514	newly 51 weeks cumul	3203.	2042.97	{1788., 2237.}	36.217	0.196969
43	newly 3 weeks spec	451.	322.08	{267., 376.}	28.5854	0.149862	newly 3 weeks cumul	1975.	1643.98	{1427., 1866.}	16.7959	0.0819028	newly 5 weeks spec	196.	106.4	{76., 140.}	45.7653	0.277047	newly 5 weeks cumul	2487.	1934.14	{1690., 2156.}	22.23	0.111061	newly 59 weeks cumul	2787.	2063.51	{1834., 2291.}	25.9595	0.132185
44	newly 3 weeks spec	403.	334.02	{276., 391.}	17.8065	0.0886114	newly 3 weeks cumul	1374.	1249.82	{1082., 1404.}	10.8399	0.0508688	newly 5 weeks spec	237.	183.13	{137., 234.}	23.5401	0.123403	newly 5 weeks cumul	1901.	1687.08	{1425., 1922.}	12.6176	0.0599854	newly 53 weeks cumul	2836.	2112.57	{1836., 2363.}	25.5088	0.13019
45	newly 3 weeks spec	487.	426.11	{251., 549.}	22.5154	0.119261	newly 3 weeks cumul	1897.	1802.25	{1303., 2125.}	15.2014	0.0721643	newly 5 weeks spec	273.	193.84	{77., 287.}	32.8498	0.216859	newly 5 weeks cumul	2573.	2285.36	{1587., 2772.}	17.3331	0.0891244	newly 56 weeks cumul	3089.	2643.95	{1696., 3295.}	20.0767	0.108432
46	newly 3 weeks spec	405.	245.77	{172., 312.}	39.316	0.235358	newly 3 weeks cumul	1271.	1010.35	{786., 1209.}	22.1424	0.115046	newly 5 weeks spec	319.	116.05	{70., 165.}	63.6207	0.469285	newly 5 weeks cumul	1984.	1301.59	{1007., 1576.}	34.4098	0.192633	newly 55 weeks cumul	2681.	1524.91	{1120., 1828.}	43.1216	0.255051
47	newly 3 weeks spec	463.	288.37	{211., 374.}	37.7171	0.21671	newly 3 weeks cumul	1416.	1149.56	{920., 1357.}	19.4887	0.0987348	newly 5 weeks spec	259.	146.98	{96., 211.}	43.251	0.275232	newly 5 weeks cumul	2039.	1507.4	{1172., 1829.}	26.1766	0.137869	newly 56 weeks cumul	2825.	1813.69	{1432., 2280.}	35.7986	0.200703
48	newly 3 weeks spec	614.	638.51	{531., 712.}	10.6075	0.0453779	newly 3 weeks cumul	1986.	2193.26	{1938., 2366.}	12.0826	0.0490986	newly 5 weeks spec	377.	396.14	{302., 454.}	14.8966	0.0641335	newly 5 weeks cumul	2819.	3104.01	{2641., 3371.}	12.9092	0.0525545	newly 53 weeks cumul	3973.	4155.25	{3328., 4565.}	13.1374	0.0563134
49	newly 3 weeks spec	412.	156.25	{94., 220.}	62.0752	0.445308	newly 3 weeks cumul	1467.	922.83	{657., 1158.}	37.0941	0.21607	newly 5 weeks spec	214.	41.52	{11., 70.}	80.5981	0.837031	newly 5 weeks cumul	2027.	1041.44	{723., 1315.}	48.6216	0.303993	newly 55 weeks cumul	2605.	1086.12	{766., 1352.}	58.3063	0.39462
50	newly 3 weeks spec	434.	284.41	{244., 329.}	34.4677	0.186689	newly 3 weeks cumul	2003.	1615.06	{1435., 1795.}	19.3679	0.0952111	newly 5 weeks spec	156.	81.51	{60., 106.}	47.75	0.293082	newly 5 weeks cumul	2450.	1849.49	{1654., 2050.}	24.5106	0.123511	newly 60 weeks cumul	2707.	1931.56	{1731., 2131.}	28.6457	0.147884
